# Prediction of the mechanical response of cardiac alternans by using an electromechanical model of human ventricular myocytes

**DOI:** 10.1186/s12938-019-0690-x

**Published:** 2019-06-07

**Authors:** Jun Ik Park, Ki Moo Lim

**Affiliations:** Department of IT Convergence Engineering, Kumoh National Institute of Technology, 61 Daehak-ro, Gumi, Gyeongbuk 39177 Republic of Korea

**Keywords:** Human ventricular myocyte, Alternans, Basic cycle length, Simulation study, Excitation–contraction coupling model

## Abstract

**Purpose:**

Although the quantitative analysis of electromechanical alternans is important, previous studies have focused on electrical alternans, and there is a lack quantitative analysis of mechanical alternans at the subcellular level according to various basic cycle lengths (BCLs). Therefore, we used the excitation–contraction (E–C) coupling model of human ventricular cells to quantitatively analyze the mechanical alternans of ventricular cells according to various BCLs.

**Methods:**

To implement E–C coupling, we used calcium transient data, which is the output data of electrical simulation using the electrophysiological model of human ventricular myocytes, as the input data of mechanical simulation using the contractile myofilament dynamics model. Moreover, we applied various loads on ventricular cells for implementation of isotonic and isometric contraction.

**Results:**

As the BCL was reduced from 1000 to 200 ms at 30 ms increments, mechanical alternans, as well as electrical alternans, were observed. At this time, the myocardial diastolic tension increased, and the contractile ATP consumption rate remained greater than zero even in the resting state. Furthermore, the time of peak tension, equivalent cell length, and contractile ATP consumption rate were all reduced. There are two tendencies that endocardial, mid-myocardial, and epicardial cells have the maximum amplitude of tension and the peak systolic tension begins to appear at a high rate under the isometric condition at a particular BCL.

**Conclusions:**

We observed mechanical alternans of ventricular myocytes as well as electrical alternans, and identified unstable conditions associated with mechanical alternans. We also determined the amount of BCL given to each ventricular cell to generate stable and high tension state in the case of isometric contraction.

## Introduction

Alternans with opposite phase is associated with electrotonic coupling and conduction velocity, as well as the appearance of a slope in the action potential duration (APD) restitution curve [1–3]. Discordant alternans is also related to arrhythmogenesis because action potentials (APs) from adjacent ventricular cells are alternating out-of-phase which amplifies repolarization gradients, promotes conduction block, and aids in re-entrant excitation [4–6]. The occurrence and complexity of re-entrant arrhythmia have a positive correlation with the number of islands of spatially discordant APD alternans [7]. Furthermore, T-wave alternans is reasonable to analyze susceptibility to ventricular arrhythmias [8, 9]. Therefore, analysis of alternans in the ventricle is very important.

At the cellular level, instabilities of membrane potential and calcium cycling can cause APD and calcium transient alternans, which are a function of pacing rate. Voltage-driven alternans shows steep APD restitution slope, and calcium-driven alternans is related to two factors: one is the release of calcium from sarcoplasmic reticulum (SR), and the other is the reduced capability of clearing calcium in cytosol. These two factors are related to pulsus alternans and T-wave alternans in heart failure [10–12]. Alternans can also arise from small fractional releases under conditions where the ability of the SR to sequester Ca^2+^ is low [13].

At the organ level, monophasic AP alternans is always related to left ventricular pressure alternans [14]. Especially, the left ventricular ejection fraction of a patient with alternans considerably decreases [15]. The mechanics of each myocyte can connect between subcellular events and ventricular activities [16].

Although quantitative analyses of electrical alternans and mechanical alternans, which can be concerned with ventricular diseases, are important, previous studies have focused on electrical alternans. It also lacks quantitative analysis of cellular mechanical alternans, which may affect organ-level ventricular mechanics according to various basic cycle lengths (BCLs). In addition, clinical studies are time-consuming and less cost-efficient. Therefore, we used an excitation–contraction (E–C) coupling model of human ventricular cells to quantitatively analyze the electromechanical alternans of ventricular cells according to a lot of BCLs (from 1000 to 200 ms at 30 ms increments).

## Methods

**Fig. 1:**
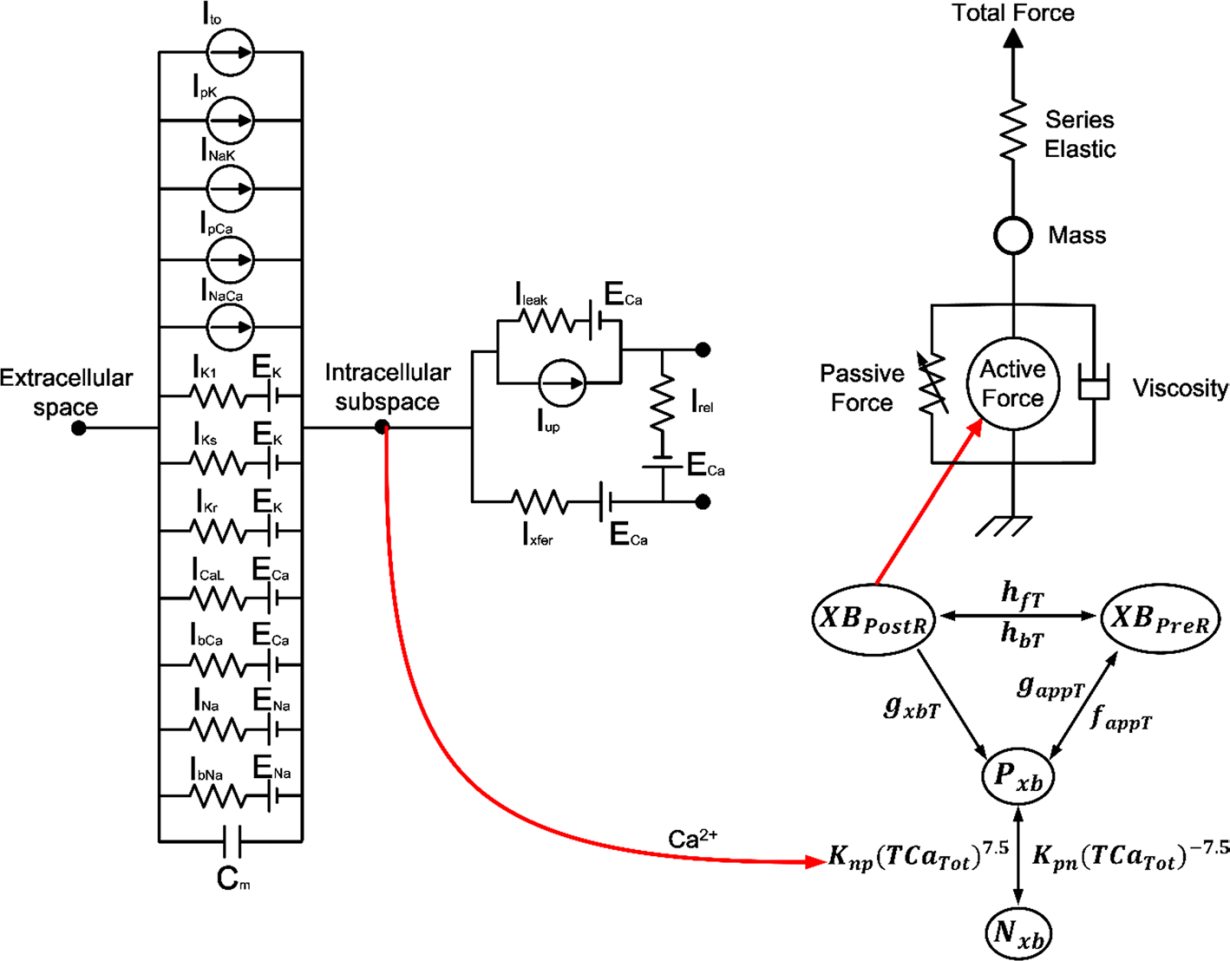
Schematic diagram of excitation–contraction coupling model of ventricular cell. The left diagram represents a human ventricular cell model with electrophysiological characteristics that mimic the ion exchange phenomenon through the cell membrane of myocytes. *I*_to_ is the transient outward K^+^ current, *I*_pK_ is the plateau K^+^ pump current, *I*_NaK_ is the Na^+^–K^+^ ion exchanger current, *I*_pCa_ is plateau Ca^2+^ pump current, and *I*_NaCa_ is the Na^+^–Ca^2+^ ion exchanger current. *E*_k_, *E*_Ca_, and *E*_Na_ are the equilibrium potentials of K^+^, Ca^2+^, and Na^+^ ions, respectively. *C*_m_ is the ventricular cell membrane capacitance in the unit surface area. *I*_K1_ is the inward rectifier K_1_ current, *I*_Ks_ is the slow delayed rectifier K^+^ current, *I*_K1_ is the rapid delayed rectifier K^+^ current, *I*_CaL_ is the L-type inward Ca^2+^ current, *I*_bCa_ denotes the background Ca^2+^ current, *I*_bNa_ is the background Na^+^ current, and *I*_Na_ is the fast inward Na^+^ current. *I*_rel_ is the release Ca^2+^ current from the sarcoplasmic reticulum (SR), *I*_leak_ is the leakage Ca^2+^ current from the SR, and *I*_up_ is the Ca^2+^ uptake current in the SR. The right diagram represents the cardiac myofilament model to simulate mechanical responses of myocytes. *N*_xb_ and *P*_xb_ are non-permissive and permissive confirmations of regulatory proteins, respectively, and XB_PreR_ and XB_PostR_ represent the probability that the cross-bridge is in the pre/post-rotated force-generating state. *g*_xbT_ is the detachment transition rate with consuming ATP, *h*_fT_ and *h*_bT_ are the forward and backward transition rates, *f*_appT_ and *g*_aapT_ are the cross-bridge attachment rate of transition and reverse rate. *K*_np_ and *K*_pn_ are transition rates for the fraction of permissive, *K*_np_(TCa_Tot_)^7.5^ is the forward rate of the non-permissive to permissive transition in the opposite direction, and *K*_pn_(TCa_Tot_)^− 7.5^ is the backward rate of the permissive to non-permissive transition. There are two types forces: active force and passive force. The active force created by contraction of the cross-bridge, and the passive force improves the complete muscle response with titin and other cytoskeletal elements. Mass prevents prompt changes in muscle-shortening velocity for quick-release protocols. Series elastic element represents effects of compliant end connections on real muscle preparations

Mechanically contracting ventricular cells exhibit the following physiological mechanisms. First, the AP of the myocytes activates voltage-activated channel in the T-tubule to release calcium from the SR into the cytosol. This causes the binding of calcium and troponin C followed by myocyte contraction cycle due to sliding of myofilaments (actin and myosin). Finally, the contractile protein movement creates cross-bridge cycling which leads to the development of active tension in the myocyte. In order to implement the contraction mechanism of the ventricular cells as described above, we recently developed human ventricular myocytes models based on three valid models [17–19]. We succeeded in quantitatively predicting the cellular mechanics by this model [20–22]. In this study, we used calcium transient obtained from the electrophysiological model of the human ventricular cells as input into the contractile myofilament dynamics model (see Fig. 1).

### Model of human ventricular cells based on electrophysiology

We used the Ten Tusscher model for human ventricular cells, which has electrophysiological characteristics consisting of a lumped parameter circuit that mimics the ion exchange phenomenon through the cell membrane of a myocyte [17, 18]. The electrophysiology behavior of a ventricular cell can be expressed by the following differential equation:

1 $$\frac{{{\text{d}}V_{\text{m}} }}{{{\text{d}}t}} = - \frac{{I_{\text{ion}} + I_{\text{stim}} }}{{C_{\text{m}} }}$$

where *V*_m_ is the cell membrane potential, *t* is the time, *I*_stim_ is the current due to external stimuli, and *C*_m_ is the capacitance of the cell membrane. *I*_ion_ denotes the sum of all transmembrane ionic currents expressed by the following equation:

2 $$\begin{aligned} I_{\text{ion}} & = I_{\text{Na}} + I_{{{\text{K}}1}} + I_{\text{to}} + I_{\text{Kr}} + I_{\text{Ks}} \\ & \quad + I_{\text{CaL}} + I_{\text{NaCa}} + I_{\text{NaK}} + I_{\text{pCa}} \\ & \quad + I_{\text{pK}} + I_{\text{bCa}} + I_{\text{bNa}}\end{aligned}$$

where *I*_Na_ is the rapid inward Na^+^ current, *I*_K1_ is the inward rectifier K^+^ current, *I*_to_ is the transient outward K^+^ current, *I*_Kr_ is the rapid delayed rectifier K^+^ current, *I*_Ks_ is the slow delayed rectifier K^+^ current, *I*_CaL_ is the L-type Ca^2+^ current, *I*_NaCa_ is the Na^+^–Ca^2+^ exchanger current, *I*_NaK_ is the Na^+^–K^+^ pump current, *I*_pCa_ is the plateau Ca^2+^ pump current, *I*_pK_ is the plateau K^+^ pump current, *I*_bCa_ is the background Ca^2+^ current, and *I*_bNa_ is the background Na^+^ current.

Calcium dynamics for the calculation of calcium transients can be described with the following equation:

3 $$I_{\text{leak}} = V_{\text{leak}} \left( {{\text{Ca}}_{\text{SR}} - {\text{Ca}}_{\text{i}} } \right)$$

4 $$I_{\text{up}} = \frac{{V_{\text{maxup}} }}{{1 + K_{\text{up}}^{2} /{\text{Ca}}_{\text{i}}^{2} }}$$

5 $$I_{\text{rel}} = V_{\text{rel}} O\left( {{\text{Ca}}_{\text{SR}} - {\text{Ca}}_{\text{ss}} } \right)$$

6 $$I_{\text{xfer}} = V_{\text{xfer}} \left( {{\text{Ca}}_{\text{SS}} - {\text{Ca}}_{\text{i}} } \right)$$

7 $$\begin{aligned} \frac{{{\text{dCa}}_{\text{itotal}} }}{{{\text{d}}t}} & = - \frac{{I_{\text{bCa}} + I_{\text{pCa}} - 2I_{\text{NaCa}} }}{{2V_{\text{C}} F}} \\ & \quad + \frac{{V_{\text{sr}} }}{{V_{\text{c}} }}\left( {I_{\text{leak}} - I_{\text{up}} } \right) + I_{\text{rel}} \end{aligned}$$

where *I*_leak_, *I*_up_, *I*_rel_, and *I*_xfer_ denote the leakage current from SR to the cytoplasm, pump current taking up calcium in SR, calcium-induced calcium-released current, and diffusive Ca^2+^ current between dyadic Ca^2+^ subspace and bulk cytoplasm, respectively. Ca_i_, Ca_sr_, and Ca_ss_ are the free cytoplasmic calcium concentration, free SR calcium concentration, and free dyadic subspace calcium concentration, respectively. *V*_leak_, *V*_maxup_, *V*_rel_, and *V*_xfer_ are the maximal *I*_leak_, *I*_up_, *I*_rel_, and *I*_xfer_, respectively. *O* is the ratio of open *I*_rel_ channels. Ca_itotal_ represents the total (free + buffered) cytoplasmic Ca^2+^ concentration. *V*_c_ and *V*_sr_ are the volume of the cytoplasm and sarcoplasmic reticulum, respectively. *F* is the Faraday constant.

We implemented each ventricular cell such as endocardial cell, mid-myocardial cell, and epicardial cell, with different conductance of ion channels. Please refer to Ten Tusscher et al. [17, 18] for details on each Eqs. (1–7).

### Model of the cardiac myofilament

We used the Rice model, which implemented the cardiac myofilament, to simulate the mechanical responses of cross-bridge cycling under isometric and isotonic contraction. The normalized active force of myocytes can be expressed by the following equation:

8 $$F_{{{\text{active}}}} \left( x \right) = {\text{SOVF}}_{{{\text{thick}}}} \left( x \right)\frac{{x{\text{XB}}_{{{\text{PreR}}}} {\text{XB}}_{{{\text{PreR}}}} + x{\text{XB}}_{{{\text{PostR}}}} {\text{XB}}_{{{\text{PostR}}}} }}{{x_{0} {\text{XB}}_{{{\text{PostR}}}}^{{{\text{Max}}}} }}$$

where *x* is the sarcomere length, ${\text{SOVF}}_{\text{thick}} \left( x \right)$ is the single-overlap fraction of the thick filament, and $x_{0}$ is the mean strain of strongly-bound state. ${\text{XB}}_{\text{PostR}}^{\text{Max}}$ are the scaling factors for state occupancy computed under optimal conditions, which indicates the fraction of strongly-bound cross-bridges. ${\text{XB}}_{\text{PreR}}$ is the probability that the cross-bridge is in the pre-rotated force-generating state while ${\text{XB}}_{\text{PostR}}$ is the probability that the cross-bridge is in the post-rotated force-generating state. The total ATP consumption, which includes strain-dependent terms, can be expressed by the following equation:

9 $${\text{ATP}} = g_{{{\text{xbT}}}} {\text{XB}}_{{{\text{PostR}}}} {\text{SOVF}}_{{{\text{thick}}}} \left( x \right)$$

where, $g_{\text{xbT}}$ is the detachment rate of the cross-bridge. The sarcomere length can be expressed by the following equation:

10 $$\frac{\text{d}}{{{\text{d}}t}}{\text{SL}} = \frac{{{\text{Integral}}_{\text{Force}} + \left( {{\text{SL}}_{0} - {\text{SL}}} \right) \times {\text{viscosity}}}}{\text{mass}}$$

where Integral_Force_, which includes preload, afterload, and passive force, denotes the total amount of normalized force over time. The *viscosity* is the viscous factor for calculating the complete muscle response. In the isosarcometric condition, $\frac{\text{d}}{{{\text{d}}t}}{\text{SL}} = 0$ and SL is constant as the initial value SL_0_. Please refer to Rice et al. [19] for details on each Eqs. (8–10).

### Simulation protocols

#### Electrical simulation

We simulated three types of human ventricular cells: endocardial cell, mid-myocardial cell, and epicardial cell. To obtain the APD restitution curve, we reduced the BCL from 1000 to 200 ms in 30 ms increments. Each cycle was repeated 30 times to obtain steady-state data, and the 29th and 30th data were used for Table 1. In the Ten Tusscher model [18], it was found that alternans occurred when the restitution slope was higher than 1. Thus, we set the APD slope as 1.8 to cause severe alternans.

**Table 1:** The quantitative values corresponding to Figs. 2, 3, 4, 5, 6

Cell type	BCL (ms)	APD (ms)	Ca^2+^ (μM)		ST (kPa)		DT (kPa)		TPT (ms)		SL
			Max	Min	IM	IT_10	IM	IT_10	IM	IT_10	IM
Endo	1000 (30th)	286	0.73	0.098	15	10	0	0	228	152	1
	280 (30th)	203	1.087	0.237	99	10	62	–	128	–	1
	280 (29th)	203	1.087	0.237	99	10	62	–	128	–	1
	200 (30th)	136	1.027	0.34	98	10	80	–	118	–	1
	200 (29th)	189	0.976	0.324	97	10	81	–	120	–	1
	600 (30th)	276	1.211	0.124	97	10	3	2	170	66	1
M	1000 (30th)	360	1.071	0.108	79	10	0	0	190	70	1
	280 (30th)	183	1.109	0.243	97	10	57	–	132	–	1
	280 (29th)	267	1.004	0.216	96	10	54	–	134	–	1
	200 (30th)	285	1.378	0.184	100	10	36	–	134	–	1
	200 (29th)	285	1.378	0.184	100	10	36	–	134	–	1
	760 (30th)	351	1.471	0.122	99	10	1	1	158	64	1
Epi	1000 (30th)	287	0.78	0.099	22	10	0	0	222	122	1
	280 (30th)	201	1.063	0.231	97	10	59	–	130	–	1
	280 (29th)	201	1.063	0.231	97	10	59	–	130	–	1
	200 (30th)	136	0.976	0.324	96	10	77	–	118	–	1
	200 (29th)	187	0.903	0.29	95	10	77	–	124	–	1
	620 (30th)	277	1.285	0.125	98	10	3	2	164	64	1

Cell type	SL		DL		TPL (ms)			ATP		TPA (ms)	
	IT_10	IT_0.6	IT_10	IT_0.6	IM	IT_10	IT_0.6	IM	IT_10	IM	IT_10
Endo	0.957	0.774	1	1	–	234	172	0.14	0.119	228	186
	0.751	0.7	0.913	0.813	–	130	116	0.913	0.419	128	78
	0.751	0.7	0.913	0.813	–	130	116	0.913	0.419	128	78
	0.769	0.71	0.863	0.786	–	122	118	0.911	0.213	118	80
	0.78	0.72	0.865	0.788	–	120	120	0.9	0.19	120	78
	0.74	0.661	1	0.960	–	170	142	0.9	0.79	170	110
M	0.789	0.683	1	1	–	170	144	0.736	0.441	190	112
	0.751	0.7	0.920	0.813	–	140	124	0.908	0.447	132	88
	0.762	0.713	0.936	0.821	–	134	118	0.89	0.446	134	82
	0.715	0.666	0.987	0.839	–	152	120	0.93	1	134	88
	0.715	0.666	0.987	0.839	–	152	120	0.93	1	134	88
	0.716	0.643	1	1	–	168	144	0.925	1.038	158	104
Epi	0.919	0.76	1	1	–	218	164	0.207	0.147	222	160
	0.754	0.704	0.923	0.816	–	132	116	0.907	0.439	130	80
	0.754	0.704	0.923	0.816	–	132	116	0.907	0.439	130	80
	0.777	0.717	0.868	0.790	–	120	116	0.899	0.204	118	76
	0.787	0.725	0.872	0.793	–	122	122	0.886	0.19	124	78
	0.731	0.654	1	0.964	–	198	140	0.912	0.877	164	106

*ST* systolic tension, *DT* diastolic tension, *TPT* time of peak tension, *SL* systolic length, *DL* diastolic length, *TPL* time of peak length, *TPA* time of peak ATP, *IM* isometric, *IT_10* isotonic (load = 10 kPa), *IT_0.6* isotonic (load = 0.6 kPa)

#### Mechanical simulation

To implement E–C coupling, we used data on calcium transient, which is the output data of electrical simulation, as the input data of mechanical simulation. We set the loads applied to ventricular cells as 10 kPa (mN/mm^2^) and 1000 kPa for implementation of isotonic contraction and isometric contraction, respectively. We also set the load as 0.6 kPa, which is the minimum value for precise calculation with our isotonic model to quantitatively determine the impact of loads under isotonic contraction.

## Results

**Fig. 2:**
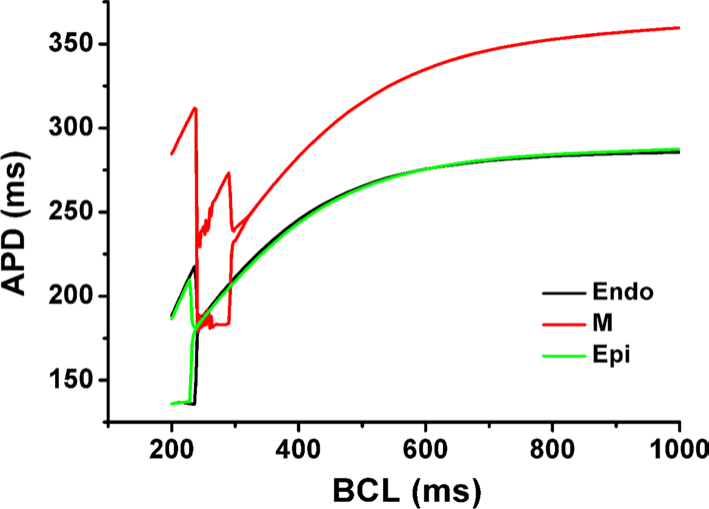
Changes in action potential duration (APD) according to basic cycle length (BCL) for each ventricular cell. Endo is the endocardial cell, M is the mid-myocardial cell, and Epi is the epicardial cell

**Fig. 3:**
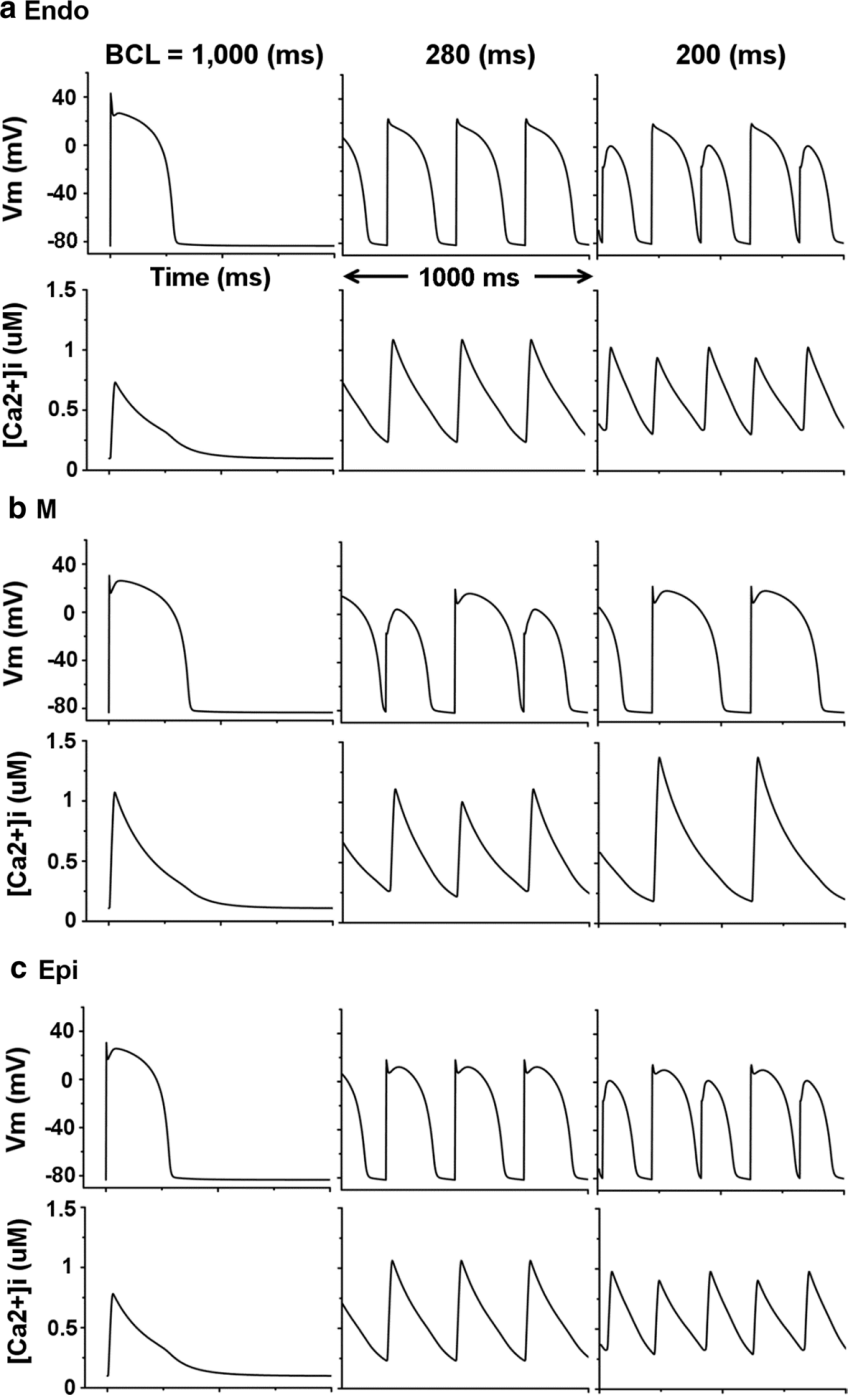
Electrical simulations of membrane potential and intracellular calcium concentration. **a**–**c** Cases of Endo, M, and Epi, respectively for 1 s. Each figure is divided into three cases according to different BCLs (1000 ms, 280 ms when electrical alternans occur under Endo and Epi conditions; and 200 ms when electrical alternans appear under M condition)

**Fig. 4:**
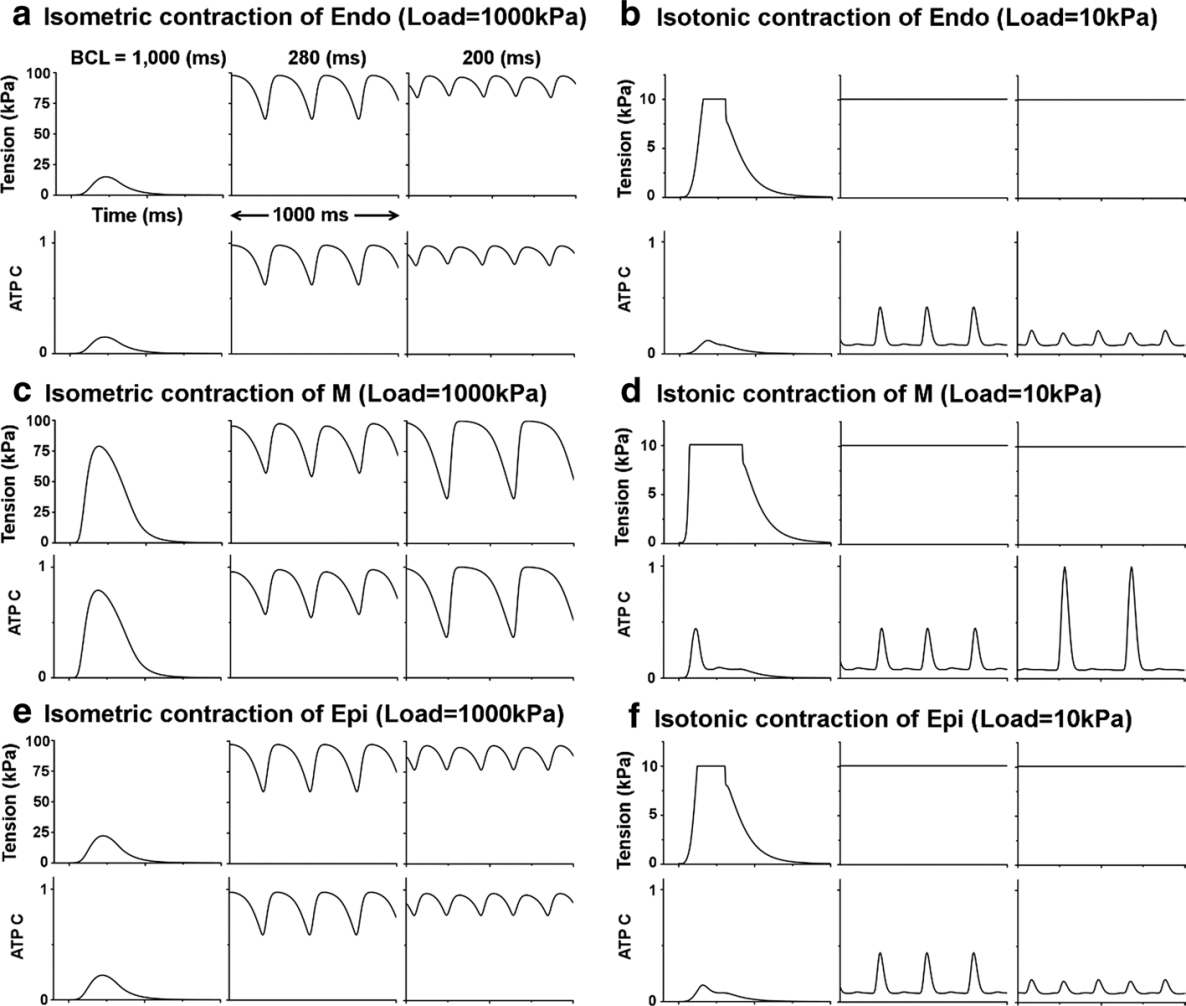
Mechanical simulations of myocardial tension and contractile ATP consumption rate. The left side represents the results of isometric contraction when the load is 1000 kPa (mN/mm^2^) and the right side shows isotonic contraction when the load is 10 kPa

**Fig. 5:**
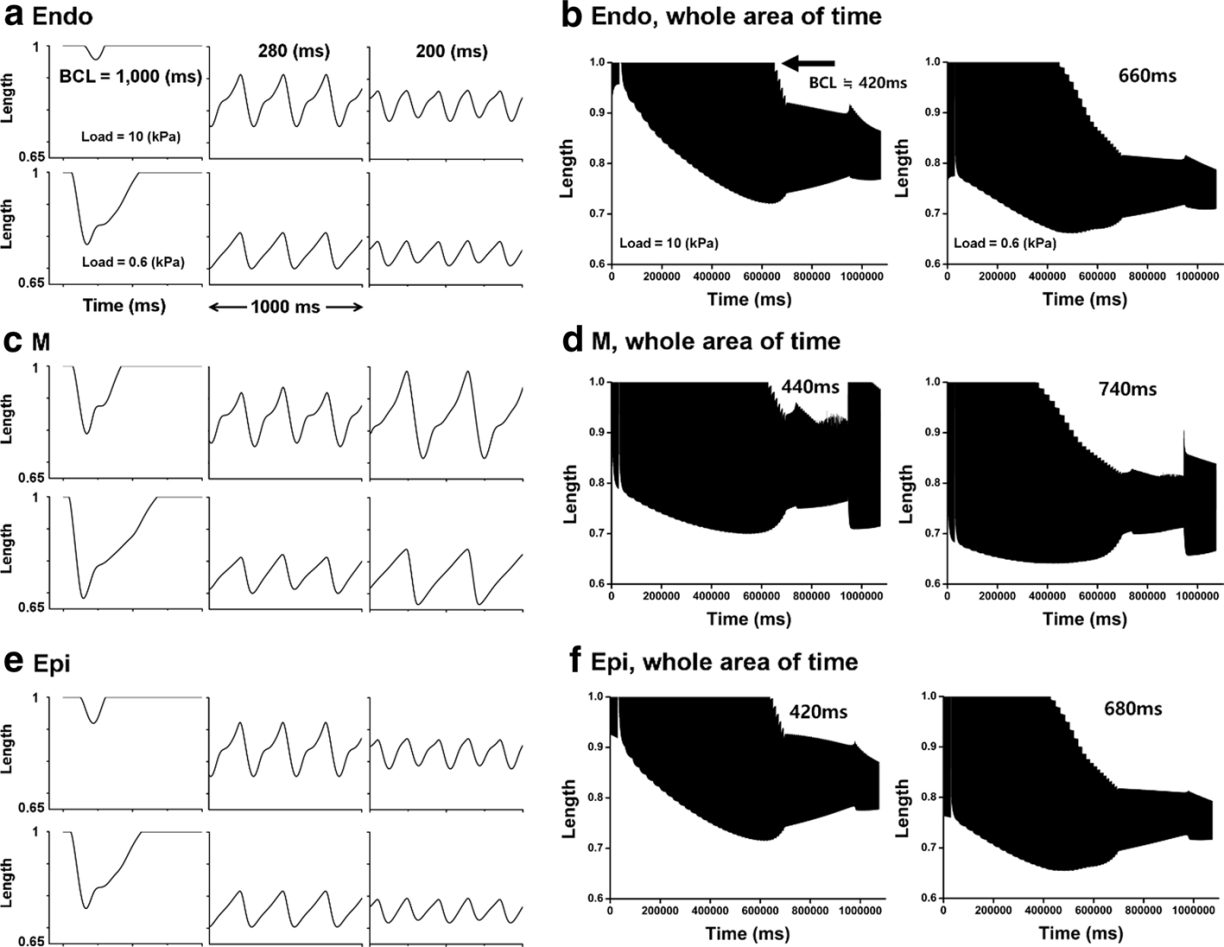
Mechanical simulations of the change in equivalent cell length in the case of three different BCLs (left) and total time from BCL of 1000 ms to 200 ms (right) when the load applied to each ventricular cell is 10 kPa and 0.6 kPa. On the right side, arrow indicates the specific BCL which takes place at the point in which the diastolic equivalent cell length is not relaxed to 1.0 (100%)

**Fig. 6:**
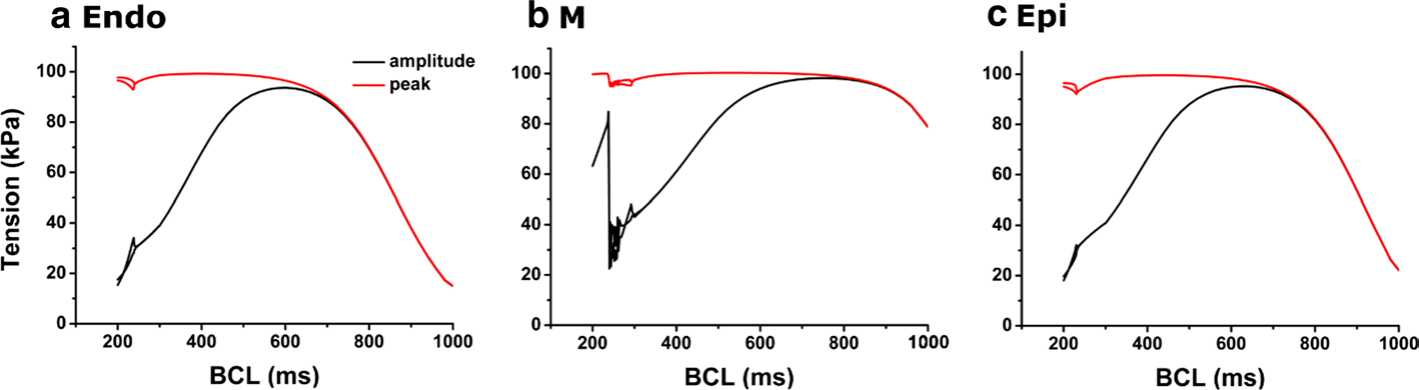
Mechanical simulations of amplitude and systolic peak of myocardial tension according to decreasing of BCL under isometric condition

The quantitative data associated with Figs. 2, 3, 4, 5, 6 are listed in Table 1.

### Results of electrical simulation according to decreasing of BCL

Figure 2 shows changes in APD with decreasing BCL from 1000 to 200 ms. The electrical alternans of endocardial cell, mid-myocardial cell, and epicardial cell started at 258 ms, 340 ms, and 244 ms, respectively. When the BCL of mid-myocardial cell decreased to 238 ms, the next stimulus was ignored by the relative refractory period so it seems like APD increased.

Figure 3 represents the membrane potential and calcium transient of endocardial cell (a), mid-myocardial cell (b), and epicardial cell (c) for 1 s according to specific BCL of ventricular cells. We set BCL as 1000 ms, 280 ms when the electrical alternans of mid-myocardial cell occurs, and 200 ms when the electrical alternans of endocardial cell and epicardial cell occur. The APD and Ca^2+^ transient at BCL of 1000 ms were larger in the order of mid-myocardial cell, epicardial cell, and endocardial cell; and discordant (out-of-phase) alternans between membrane potential and Ca^2+^ transient was observed in all three ventricular cells (Fig. 3a–c). As BCL decreased, Ca^2+^ transient increased (Fig. 3a, c). As shown in Fig. 2, when the BCL of mid-myocardial cell is 200 ms, the APD and maximum Ca^2+^ transient increased compared with BCL of 280 ms (Fig. 3b). In addition, we found that the electrical alternans of ventricular cells had APD/BCL higher than 0.9 but less than 1 (Fig. 3 and Table 1).

### Results of mechanical simulation as decreasing of BCL

Figure 4 illustrates the result of mechanical simulation using the Ca^2+^ transient, which is the output data from electrical simulation. Myocardial tension and contractile ATP consumption rate were observed/found under isometric and isotonic conditions. Endocardial cell, mid-myocardial cell, and epicardial cell were compared to determine the maximum ATP consumption rate; then, we normalized the maximum value as 1 (see Fig. 4d). Under isometric condition (Fig. 4a, c, e), the systolic tension was larger and the time of peak systolic tension was faster in the order of mid-myocardial cell, epicardial cell, and endocardial cell, when BCL was 1000 ms. Specific BCL (mid-myocardial cell: 280 ms, endocardial cell, and epicardial cell: 200 ms), when occurred electrical and Ca^2+^ transient alternans (Fig. 3), also showed mechanical alternans, which means alternans of myocardial tension and contractile ATP consumption. Under isotonic condition (Fig. 4b, d, f), the time of peak systolic tension was fast and the duration of systolic tension was long in the order of mid-myocardial cell, epicardial cell, and endocardial cell when BCL was 1000 ms. When the BCL of all ventricular cells was 280 and 200 ms, systolic tension was constant as the load value regardless of the Ca^2+^ transient and contractile ATP consumption rates. When the BCL of mid-myocardial cell was 200 ms, the amplitude of myocardial tension and contractile ATP consumption rate increased, and the refractory period of the cardiac cycle became longer (Fig. 4c, d). Overall, the maximum contractile ATP consumption rate decreased and the minimum contractile ATP consumption rate was greater than 0 when mechanical alternans occurred.

Figure 5 shows the equivalent cell length of isotonic contraction under 10 kPa and 0.6 kPa conditions at three different BCLs (Fig. 5a, c, e) as well as the total time from BCL of 1000 ms to 200 ms (Fig. 5b, d, f). In all three ventricular cells, systolic length was short in the order of mid-myocardial cell, epicardial cell, and endocardial cell regardless of load. Diastolic length was not relaxed to 1 (100%) when BCL was 280 and 200 ms (Fig. 5a, c, e). When the load of ventricle cells was 0.6 kPa compared with the 10 kPa condition as BCL decreased, the specific BCL (arrow in Fig. 5b, d, f), which showed the starting point at which diastolic length was not relaxed to 1 was higher and the systolic length was always shorter (Fig. 5b, d, f).

Figure 6 shows the amplitude and maximum value of myocardial tension with decreasing BCL under isometric condition. As BCL decreased, the amplitude of tension and the peak of systolic tension increased. When the BCL of endocardial cell, mid-myocardial cell, and epicardial cell exceeded 600, 760 and 620 ms, respectively, the amplitude was highest and then decreased, and the peak value remained high. The timing of alternans in myocardial tension was the same as when electrical alternans occurred (Fig. 2).

## Discussion

In the present study, we used an excitation–contraction coupling model of human ventricular cells to quantitatively analyze the electromechanical responses with discordant electromechanical alternans according to decreasing of BCL from 1000 to 200 ms. The main findings are as follows:

1. As the BCL of the ventricular myocytes decreased, the out-of-phase (discordant) alternans between membrane potential and Ca^2+^ transient occurred in the order of mid-myocardial cell, endocardial cell, and epicardial cell. Mechanical alternans such as alternans of myocardial tension, contractile ATP rate, and equivalent cell length were also observed (Figs. 2, 3, 4, 5, 6 and Table 1).
2. As the BCL of ventricular cells decreased, the minimum Ca^2+^ transient increased simultaneously with the occurrence/emergence of the electromechanical alternans of ventricular cells. Moreover, myocardial diastolic tension also increased and contractile ATP consumption rate was greater than 0 under isometric condition (Figs. 2, 3, 4 and Table 1).
3. When mechanical alternans occurred (BCL = 200 ms) under isotonic contraction, the variations in sarcomere length and contractile ATP consumption rate were smaller than in the case of no alternans (BCL = 280 ms), except the mid-myocardial cell cell (Figs. 4, 5 and Table 1).
4. From a specific BCL (arrow in Fig. 5b, d, f), the equivalent cell length was not relaxed to 1 (100%) and the systolic tension was always fixed at the same value as the load under isotonic condition. When the load was low, specific BCLs appeared faster and systolic length was always shorter (Figs. 4, 5 and Table 1).
5. Under isometric condition with decreasing BCL, the amplitude of tension was largest at BCL of 600 ms (endocardial cell), 760 ms (mid-myocardial cell), and 620 ms (epicardial cell), and the peak of systolic tension began to appear at a high rate (Fig. 6 and Table 1).

Ventricular tissue consists of three tissue structures, namely, endocardium, mid-myocardium, and epicardium. These tissues have different electrophysiological characteristics based on their structures [23]. For example, endocardium, the outermost layer of the heart, provides essential signals, such as Hedgehog signal for the continued growth and differentiation of the heart [24] and regulates the neighboring cardiac outflow tract [25, 26]. In addition, the activation of growth factors and trans-differentiation of fibroblasts and immunocytochemical characterizations vary according to the endocardium, mid-myocardium, and epicardium [27]. microRNA is involved in the regeneration of epicardium from cardiovascular disorders [28–30]. The adult cardiac stem cells are multipotent and participate in the regeneration of mid-myocardium [31, 32]. Therefore, it is necessary to observe the mechanical response of cardiac alternans in the endocardial cell, mid-myocardial, and epicardial cells of the ventricular tissue, respectively.

The electrical instability of ventricular cells led to mechanical instability because mechanical alternans such as alternans of myocardial tension, contractile ATP rate, and equivalent cell length also occurred when BCL was 280 ms and 200 ms, which was same timing as when discordant electrical alternans occurred (Figs. 2, 3, 4, 5, 6 and Table 1). Eventually, this will lead to the failure of ventricular mechanics at the organ level; for instance, a decrease in left ventricular ejection fraction [14–16].

In myocardial tissue cells with reduced BCL below a certain level, alternans are more likely to develop than in the normal BCL cells. This is because, the myocardial cells with short BCL do not have enough refractory period and diastolic interval as shown in Fig. 2 (refer [1, 2]). When a discordant electrical alternans occur, a reduced diastolic interval lowers the cytosolic Ca^2+^ sequestration. This results in elevated free Ca^2+^ concentration in the cytosol (Fig. 3 and Table 1). This is again consistent with the fact that reduced capability of Ca^2+^ sequestration in the cytosol is related to the development of alternans [10, 12].

Myocardial diastolic tension increased with elevated minimum Ca^2+^ transient. When myocardial cell with normal BCL was contracted, rate of contractile ATP consumption is 0 during resting state. However, in case of mechanical alternans, contractile ATP consumption rate is always greater than 0 even resting state. This suggests that when mechanical alternans occurs at low BCL, the unstable state of ventricular cells can occur because of the continuous detachment of the cross-bridge (Fig. 4 and Table 1).

In endocardial and epicardial cells, reduced Ca^2+^ transient due to mechanical alternans lowers contractile ATP consumption rate as compared to the state without mechanical alternans. It results in a reduction of the difference between the systolic and diastolic length (Figs. 4b, f, 5a, e). Similar to endocardial cell and epicardial cell, mid-myocardial cell also has smaller changes in equivalent cell length and contractile ATP consumption rate compared with before the occurrence of electromechanical alternans (BCL > 340 ms in Fig. 2). However, this was not shown in the graph to avoid complexity.

Under isotonic contraction, the ventricular cells from the specific BCL (marked by arrows in Fig. 5b, d, f) are not completely relaxed, in which the equivalent length of the cells is not one. It is because the myosin head always forms a cross-bridge even with a higher load regardless of the sarcomere length without double overlap of the thin filament. Therefore, the systolic tension is the same as that of the load (Figs. 4, 5 and Table 1). During isotonic contraction, the lighter is the load of the ventricular cells, the lower is the rate of cross-bridge formation and the greater is the minimum BCL at which the cells are completely relaxed. Hence, when the load of the ventricular cells was 0.6 kPa during isotonic contraction, the minimum BCL was longer than 10 kPa load (marked by arrows in Fig. 5b, d, f). Thus, if the load of ventricular cells is low under isotonic condition, the unstable state occurs as BCL decreased. In addition, the change in sarcomere length is inversely proportional to the load (see Eq. 10). Thus, when load of ventricular cells is small, the systolic equivalent cell length is shorter than under the condition of high load (Fig. 5 and Table 1).

Under isometric condition, the peak systolic tension remained high and the amplitude of tension was highest at the specific BCL without mechanical alternans. It is possible to predict the amount of BCL given to each ventricular cell to generate the most efficient and stable tension (Fig. 6 and Table 1).

### Limitations

This study has several limitations. First, experimental or clinical data were not collected as part of this study. Instead, the validated ventricular cell model and methodologies from previous studies were applied, such as the model of human ventricular cells [17, 18] and myofilament dynamics [19]. Second, we used the one-way EC coupling model to prevent the mechanical activity of ventricular cells from affecting the electrophysiological behavior of the heart, although such phenomena could occur physiologically. Finally, we simplified only the three-state cross-bridge cycle using the Rice model [19], but there are many more states in biochemical studies. In order to overcome the limitations, we will need to have more clinical data for validation of the model and apply more sophisticated numerical method for full E–C coupling.

## Conclusion

As the BCL decreased, mechanical alternans (myocardial tension, contractile ATP consumption rate, and equivalent cell length) occurred as well as out-of-phase alternans between membrane potential and Ca^2+^. Simultaneously, myocardial diastolic tension increased and contractile ATP consumption rate remained greater than 0. Moreover, the time of peak tension, equivalent cell length, and contractile ATP rate decreased. Under isotonic condition, when electromechanical alternans occurred, the changes in sarcomere length and contractile ATP consumption rate were reduced compared with before the occurrence of alternans. The unstable state, in which cross-bridge formation rate was the same as the load regardless of sarcomere length, appeared quickly when the load of ventricular cells was low. In addition, we can see the amount of BCL given to each ventricular cell to generate stable and high tension states in the case of isometric contraction.

## Data Availability

Not applicable.
